# Manipulation of Antiferromagnetic Metal Phase in Nd_1‐x_Ce_x_NiO_3_ by Epitaxial Strain

**DOI:** 10.1002/advs.202415785

**Published:** 2025-03-16

**Authors:** Zhan Yang, Junhua Liu, Wen Xiao, Shilin Hu, Zhixiong Deng, Xuedong Bai, Lei liao, Yulin Gan, Kai Chen, Lifen Wang, Zhaoliang Liao, Haizhong Guo

**Affiliations:** ^1^ School of Physics Zhengzhou University Zhengzhou 450001 China; ^2^ National Synchrotron Radiation Laboratory School of Nuclear Science and Technology University of Science and Technology of China Hefei 230029 China; ^3^ Beijing National Laboratory for Condensed Matter Physics and Institute of Physics Chinese Academy of Sciences Beijing 100190 China; ^4^ School of Physical Sciences University of Chinese Academy of Sciences Beijing 100049 China; ^5^ Institute of Quantum Materials and Physics Henan Academy of Sciences Zhengzhou 450046 China

**Keywords:** antiferromagnetic metals (AFMs), epitaxial strain, metal–metal transition temperature, Néel temperature, structural transition

## Abstract

Antiferromagnetic metals (AFMs) are potential candidates for spintronics application owing to their insensitivity to external magnetic perturbations. However, the scarcity of AFM in complex oxide presents a significant challenge in tuning their critical properties, thereby impeding the exploration of emergent phenomena and the advancement of practical applications. Quite recently, an AFM ground state is discovered in Nd_1‐x_Ce_x_NiO_3_, an oxide whose undoped parent counterpart exhibits metal‐insulator transition dependent on temperature. Herein, the engineering of the AFM state by epitaxial strain in Nd_1‐x_Ce_x_NiO_3_ (0 ≤ x ≤ 0.07) films is demonstrated, where both Néel temperature and the metal–metal transition temperature exhibit significant response. Particularly in the 5% Ce‐doping counterpart (Nd_0.95_Ce_0.05_NiO_3_), a suppression of the structural transition driven by compression strain causes a transition of the electronic/magnetic ground state from the AFM to paramagnetic metal. The O‐K edge X‐ray absorption spectra (XAS) reveal that strain plays a crucial role in modulating the magnetic ground state through modifying Ni─O hybridization. This work demonstrates the successful engineering of the electronic/magnetic states of AFM through epitaxial strain, providing a vital roadmap for the development of nickelate‐based AFM devices.

## Graphical abstract

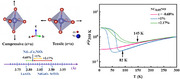

## 1 Introduction

The discovery of infinite‐layer Ni‐based superconductors has sparked a reexamination of rare‐earth nickelate perovskite *RE*NiO_3_.^[^ ^1^, ^2^, ^3^, ^4^ ^]^ The strong electron correlation effects arising from the localized Ni 3d orbitals lead *RE*NiO_3_ (*RE* ≠ La) both in bulk and films to undergo a metal‐insulator transition (MIT) and paramagnetic‐antiferromagnetic transition as temperature decreases,^[^ ^5^, ^6^, ^7^, ^8^, ^9^ ^]^ along with exhibiting strain and dimension effects.^[^ ^10^, ^11^ ^]^ Among them, NdNiO_3_ as one of its rare‐earth nickelate counterparts, straddling the boundary between localized and itinerant electron behaviors, has been the focus of study due to its various physical phenomena, such as both spin/charge ordering,^[^ ^12^, ^13^, ^14^, ^15^ ^]^ thermochromic effect^[^ ^16^ ^]^ and a transition from metallic paramagnetic to insulating antiferromagnetic state occurring concurrently with charge^[^ ^6^, ^17^, ^18^ ^]^ or bond^[^ ^19^ ^]^ disproportionation ∼200 K. Several reports have revealed that chemical substitution with hole doping (e.g., A‐site Sr doping) modifies the electronic structure of NdNiO_3_ films.^[^ ^20^, ^21^, ^22^ ^]^ On one hand, the introduction of holes can significantly suppress antiferromagnetic order, leading to a paramagnetic metal ground state akin to the correlated metal LaNiO_3_ films,^[^ ^4^, ^21^, ^23^, ^24^ ^]^ such as Nd_1‐x_Sr_x_NiO_3_ films, a precursor phase of Ni‐based superconductor that has recently garnered attention;^[^ ^1^, ^2^ ^]^ On the other hand, hole doping also inhibits the MIT, thereby shifting the transition point to lower temperatures.^[^ ^21^ ^]^ Intriguingly, the observation of the rare antiferromagnetic metal (AFM) ground state in electron‐doped NdNiO_3_ films through Ce substitution (Nd_1‐x_Ce_x_NiO_3_), as reported very recently,^[^ ^9^ ^]^ has injected new vitality into the field of nickelates. Especially, the robustness against external field perturbations, absence of stray fields, ultrafast dynamics, and potential for generating large magnetotransport effects of the AFM endows promise for future spintronics.^[^ ^25^, ^26^, ^27^, ^28^ ^]^ Engineering this AFM state is expected to provide crucial insights for the design of magnetics and electronics of nickelates, as well as for potential device applications.

The rich interplay and intertwining of spin, orbital, charge, and lattice degrees of freedom in complex transition metal oxides not only give rise to a plethora of intriguing physical properties but also result in significant coupling of these properties with external physical fields. Among these, the strain field, as one of the most readily accessible means of manipulating electronic structure, has played a significant role in the control of properties in perovskite oxides.^[^ ^29^, ^30^, ^31^, ^32^, ^33^ ^]^ Generally, the fundamental step in employing heteroepitaxial engineering is to modify, through orbital‐lattice interactions, the d orbital population and overlapping driven by strain‐induced lattice deformations.^[^ ^34^, ^35^, ^36^, ^37^ ^]^ Particularly in rare‐earth nickelate films *RE*NiO_3_, epitaxial strain can directly alter the e_g_ bandwidth by modulating the hybridization between Ni 3d and O 2p orbitals, stemming from the strain‐induced modifications of Ni─O bond lengths and [NiO_6_] octahedral tilts (or Ni─O─Ni bond angles).^[^ ^38^ ^]^ This can, in turn, regulate the negative charge‐transfer gap between the O 2p band and Ni 3d upper Hubbard band, thereby influencing the corresponding MIT and accompanying magnetic transition.^[^ ^17^ ^]^ Therefore, epitaxial strain holds potential as a promising avenue for manipulating the AFM state in the electron‐doped counterparts of perovskite nickelates Nd_1‐x_Ce_x_NiO_3_.

Here, we systematically investigated the engineering of the antiferromagnetic metallic ground state in the electron‐doped Nd_1‐x_Ce_x_NiO_3_ films (≈15 nm) by epitaxial strain offered by varied substrates. The introduction of Ce^4+^ ions altered the band structure and geometry distortions, resulting in a concentration dependence of the transition from paramagnetic metal to antiferromagnetic metal. In particular, the lattice strain significantly modified the in‐plane Ni─O bond lengths and out‐of‐plane Ni─O─Ni bond angles, demonstrating a substantial control over the magnetic ground state. Ultimately, compressive strain induced the ground‐state transition from antiferromagnetic metal to paramagnetic metal in Nd_0.95_Ce_0.05_NiO_3_. XAS further revealed the correlation among strain‐driven structural distortions, Ni─O hybridization, and the magnetic ground state. This work is not only a successful attempt at artificially engineering the AFM state in complex oxide, but it also further emphasizes the key role of strain in regulating the electronic and magnetic structures of nickelates.

## 2 Results and Discussion

**Figure 1:**
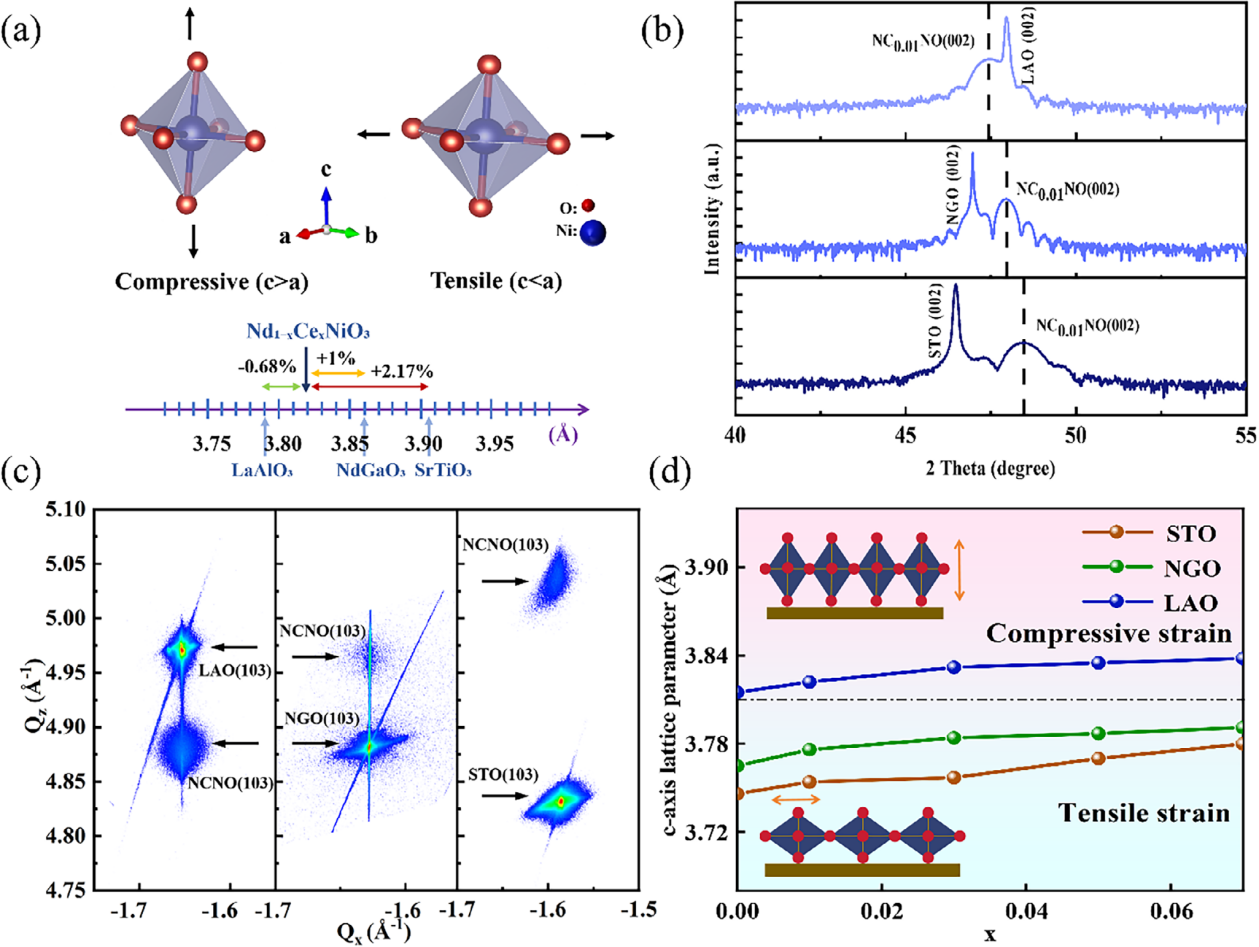
a) The scheme of biaxial strain states of the Nd_1‐x_Ce_x_NiO_3_ films grown on LAO, NGO, and STO substrates. b) X‐ray diffraction patterns of the Nd_0.99_Ce_0.01_NiO_3_ films grown on varied substrates. c) Reciprocal space mapping (RSM) results of Nd_0.99_Ce_0.01_NiO_3_ films. d) *c*‐axis lattice parameters as functions of Ce^4+^ content, which is extracted and calculated from Figure S3 (Supporting Information). The insets show the lattice distortion under different strains, respectively.

High‐quality Nd_1‐x_Ce_x_NiO_3_ (NC_X_NO, x = 0, 0.01, 0.03, 0.05, 0.07) films were rationally grown on different substrates, including SrTiO_3_ (001)_c_, LaAlO_3_ (001)_pc_ and NdGaO_3_ (001)_pc_ by pulsed laser deposition, where c and pc denote cubic and pseudocubic structures, respectively. The thickness of the films was controlled in situ by monitoring the reflecting high‐energy electron diffraction (RHEED) intensity oscillations, in which the layer‐by‐layer growth fashion arises the atomically smooth surface, as shown in Figure S1 (Supporting Information). The clear Kiessig fringes shown in the X‐ray reflectivity (XRR) curve also indicate that the sample possesses a smooth interface and surface. The simulated results derived from XRR show that the sample thickness is ≈15 nm (see Figure S2, Supporting Information), which is consistent with the RHEED oscillation. The bulk Nd_1‐x_Ce_x_NiO_3_ (0.01 ≤ x ≤ 0.07) under pseudocubic scenario exhibits lattice constant of ≈3.82 Å,^[^ ^9^ ^]^ therefore, the substrates of STO (001)_c_, NGO (001)_pc_, and LAO (001)_pc_ can provide ≈2.17%, 1% tensile strain, and 0.68% compressive strain, respectively (see **Figure** **1**a).

Figure 1b shows an X‐ray diffraction (XRD) pattern around (002) peak of Nd_0.99_Ce_0.01_NiO_3_ films grown on various substrates. The presence of only (002) diffraction peak, along with clear Laue oscillation, indicates epitaxial characteristics and the smooth surface. The full‐width‐of‐half‐maximum (FWHM) of the rocking curve around the (002) peak is as low as 0.017° (See Figure S3, Supporting Information), highlighting the excellent crystallinity of the films. According to the RSM of different samples, the aligned Q_x_ values between substrate and film confirm the coherent growth (see Figure 1c). Consequently, under compressive (LAO) or tensile (NGO, STO) strain, the out‐of‐plane lattice constant of the film exhibits expansion or compression, respectively, as illustrated in Figure 1d. Notably, although the Ce^4+^ (0.87 Å) radius is smaller than that of Nd^3+^(0.983 Å),^[^ ^39^ ^]^ the generated Ni^2+^ increase arising from Ce^4+^ substituting Nd^3+^ leads to the *c*‐axis expansion due to its much larger ions radius than Ni^3+^. Therefore, the out‐of‐plane lattice parameter increases as elevating the Ce^4+^ doping concentration constantly.

**Figure 2:**
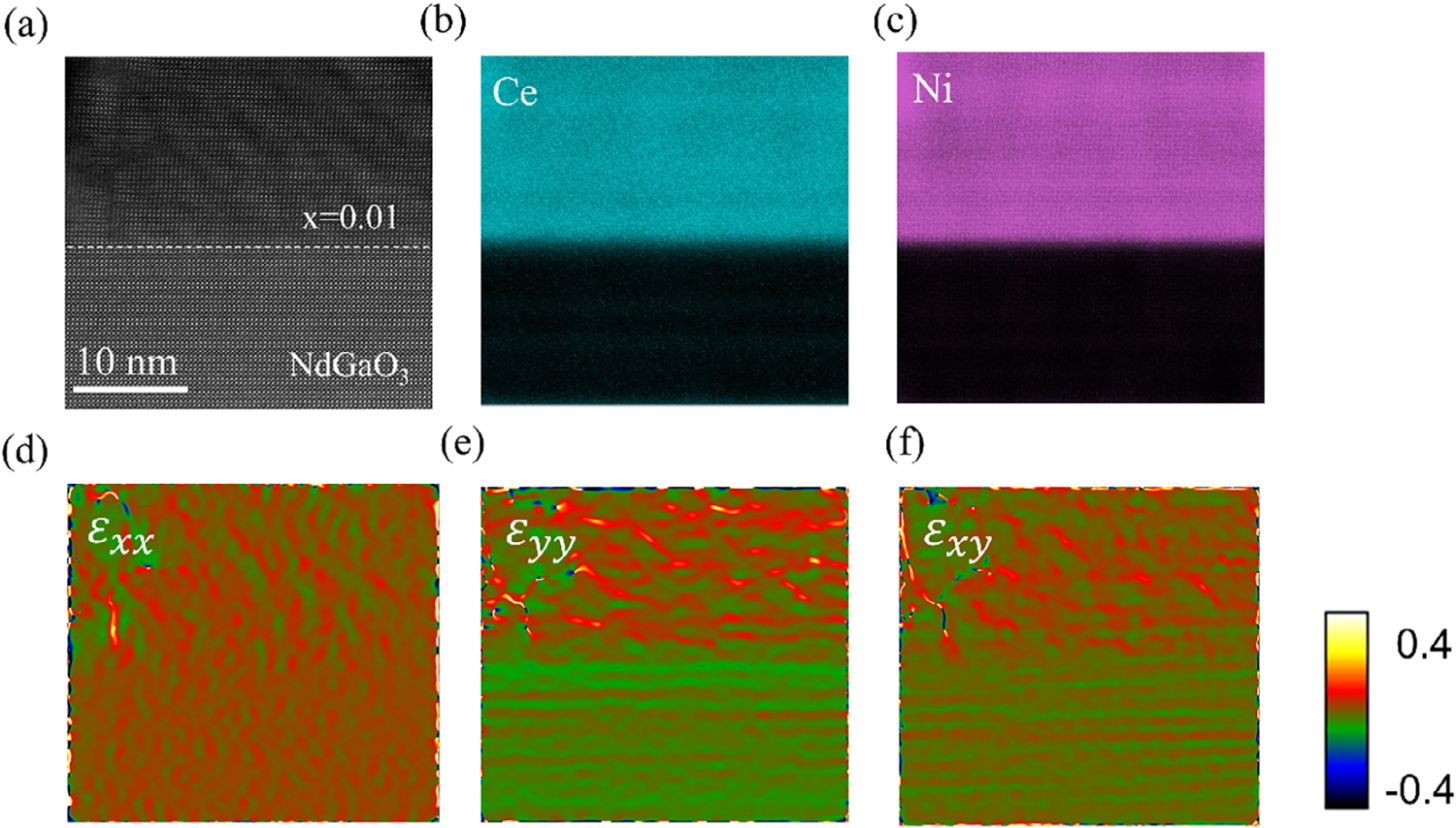
a) HAADF image of the Nd_1−x_Ce_x_NiO_3_/NGO (x = 0.01) heterostructure along [100]_pc_ direction. b,c) Corresponding EDS mappings of Ce (M edge) and Ni (K edge) elements. d–f) Corresponding GPA for reflecting strain distributions ε_xx_, ε_yy,_ and ε_xy_.

To elucidate the uniformity of Ce distribution in films, scanning transmission electron microscopy (STEM) measurement of Nd_0.99_Ce_0.01_NiO_3_ film is conducted. The high‐angle annular dark field (HAADF) image reveals periodic atomic arrangement and thus excellent crystallinity in the film (see **Figure** **2**a). The global EDS mapping of the Ce and Ni elements was shown in Figure 2b,c, respectively, in which their uniform dispersion dispels concerns about potential changes in physical properties caused by Ce aggregation. Furthermore, the strain state of the film is determined by geometric phase analysis (GPA) of the HAADF image. Among them, the in‐plane strain ε_xx_ exhibits almost uniform distribution across the entire heterostructure as shown in Figure 2d, reflecting the epitaxial coherence of the film. For the out‐of‐plane strain ε_yy_ and shear strain ε_xy_, some discernable strain contrast can be observed, which can be attributed to lattice distortion induced by the in‐plane constraint of the substrate. These results further are consistent with the RSM shown in Figure 1c, highlighting the effective propagation of the substrate strain.

**Figure 3:**
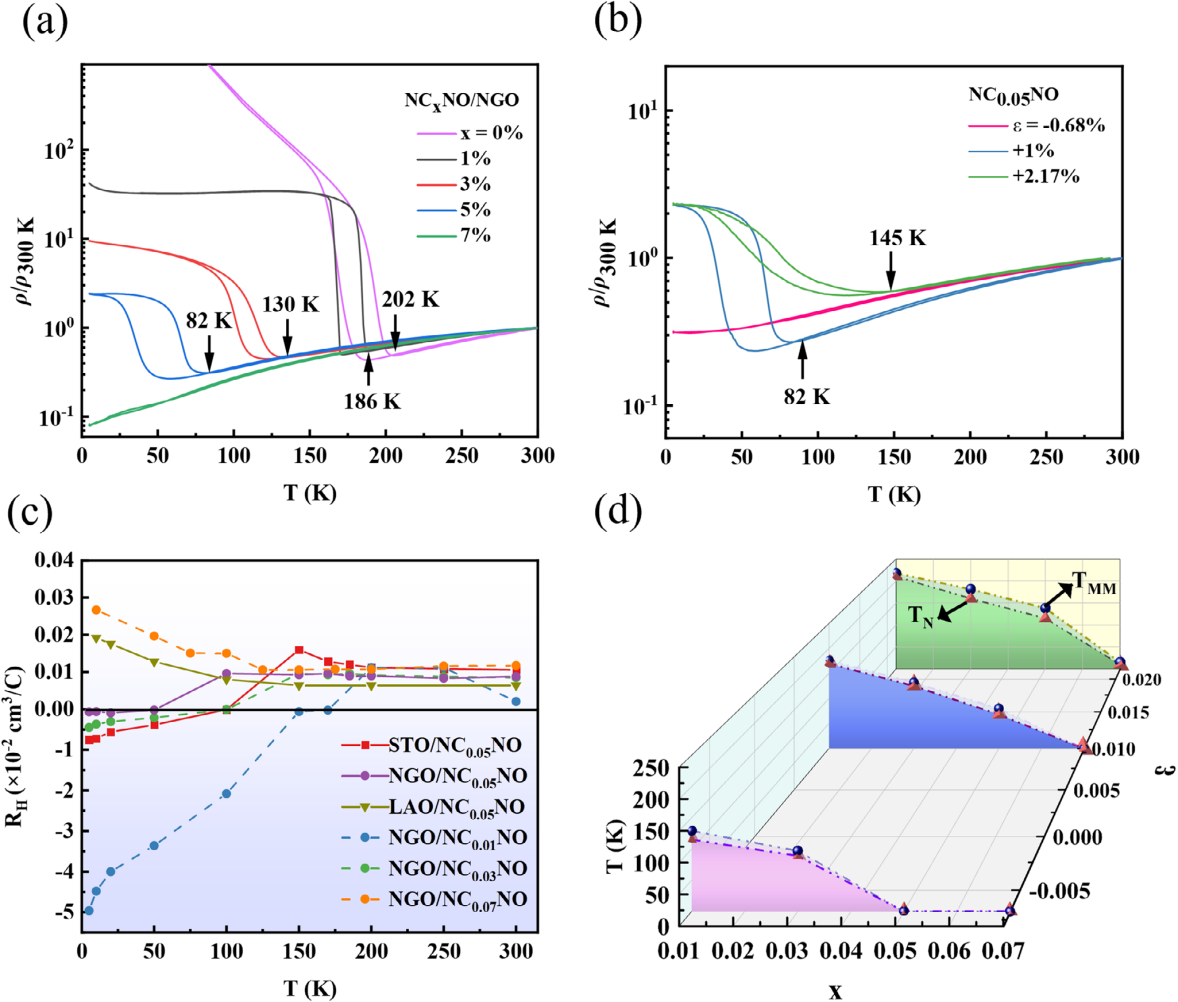
a) Temperature‐dependent resistivity (*ρ*‐T) curves of Nd_1‐x_Ce_x_NiO_3_ (0 ≤ x ≤ 0.07) films grown on NGO. b) *ρ*‐T curves of Nd_1‐x_Ce_x_NiO_3_ (x = 0.05) under different strain (*ε*). c) Temperature dependence of Hall coefficient of different films. d) Strain (*ε*) dependence of T_N_ and T_MM_ under different doping (x).

The antiferromagnetism and metallicity of NC_X_NO are demonstrated through transport measurements. In **Figure** **3**a, the undoped NNO exhibits a typical paramagnetic metal to antiferromagnetic insulator transition around T = 200 K as the temperature decreases, consistent with previous reports.^[^ ^40^, ^41^ ^]^ By introducing donor dopant (A‐site Ce^4+^ substitution), additional conduction channels are activated^[^ ^9^ ^]^ so as to switches the MIT into a metal–metal transition (MMT) picture, eventually leading to the metallic ground state of NC_X_NO as shown in Figure 3a. An additional epitaxial ferromagnetic La_2/3_Sr_1/3_MnO_3_ (15 uc) was capped on NC_X_NO film for verifying its magnetism (see Figure S4a, Supporting Information), where the observed significant exchange bias collaborating with its metallicity evidences the antiferromagnetic metal ground state in NC_X_NO. In fact, whether growing on tensile‐strained STO or compressive‐strained LAO substrates, transport characterization of samples exhibits a similar evolution (Figure S5c,d, Supporting Information) with doping level. Especially, electronically driven carrier injection induced by Ce doping can lower the antiferromagnetic ordering temperature,^[^ ^6^, ^40^, ^42^, ^43^ ^]^ eventually forming a paramagnetic metallic state (NC_0.07_NO) similar to the rare earth La counterpart LaNiO_3._^[^ ^44^, ^45^ ^]^

Then, taking 5% Ce‐doped samples (Nd_0.95_Ce_0.05_NiO_3_) on different substrates for example, the strain‐induced regulation of MMT was investigated. As shown in Figure 3b, the MMT temperature (T_MM_) decreases as a switching from tensile to compressive strain. This effect can be attributed to the suppression of lattice distortions in the nickelate, with the primary cause in this instance being the applied strain, rather than the increased Ce^4+^ doping discussed above. Of particular note is the disappearance of the T_MM_ under the compressive strain provided by the LAO substrate, and it seems to mean the vanishing of the paramagnetic‐antiferromagnetic transition as well. This observation suggests that the external strain exerted by the substrate can significantly modulate both the metal–metal transition and the magnetic ground state.

The ordinary Hall coefficient (R_H_) is a useful physical quantity for studying changes in the electronic structure. As shown in Figure 3c, similar to the undoped NNO film,^[^ ^46^ ^]^ the temperature‐driven MIT is accompanied by a sign switching of R_H_. In the metallic‐state temperature range, transport is determined by both a large hole pocket and a small electron pocket near the Fermi surface, resulting in an overall hole‐type behavior (R_H_ > 0).^[^ ^47^, ^48^ ^]^ The temperature‐driven transition from orthorhombic to monoclinic phase triggers the gap opening in the hole bands, leading to residual electron‐type carrier transport in the low‐temperature insulating state (R_H_ < 0).^[^ ^46^ ^]^ Although the density of states is suppressed at low temperatures, the band reconstructing induced by donor dopant Ce^4+^ results in finite values of the density of states near certain momenta, thereby allowing for metallic characteristics at low temperatures.^[^ ^9^ ^]^ Interestingly, increasing the Ce^4+^ concentration leads to a decrease or even disappearance of the temperature at which R_H_ changes from positive to negative, indicating the suppression of the gap opening in the hole bands, in line with the results shown in Figure 3a.

To investigate the Néel temperature detailly, the angle‐dependent AMR measurement of NC_X_NO films under different doping and strain levels (see Figure S4c, Supporting Information) was demonstrated, where the results are summarized in Figure 3d. The applying of tensile strain shifts the antiferromagnetic transition point to a higher temperature. Conversely, the compressive strain has an inhibitory effect on the antiferromagnetism, making the magnetic order more susceptible to fluctuation‐induced destruction and thereby exhibiting a lower Néel temperature or even completely suppressing the antiferromagnetic order, e.g. the 5% doped counterpart NC_0.05_NO. Eventually, it shows a paramagnetic ground state on the LAO substrate, similar to the phenomenon in the parent phase NNO.^[^ ^49^ ^]^ In fact, strain effects directly modulate the buckling of the oxygen octahedra (i.e., buckling angle) and the Ni─O bond length, allowing the entire crystal structure to adapt to the substrate. The Ni─O bond is more compressible than the distance between O^2−^ and A‐site ions.^[^ ^50^ ^]^ Therefore, the compressive strain favors to shorten in‐plane Ni─O bond length, resulting in a possible straightening of out‐of‐plane Ni─O─Ni buckling angle.^[^ ^51^ ^]^ Consequently, NC_X_NO under compressive strain (i.e., grown on LAO substrate) tends toward an orthorhombic structure, leading to the absence of a temperature‐driven structural phase transition and consistently exhibiting a hole‐dominated metallic state (R_H_ > 0).

Furthermore, the MMT temperature T_MM_ exhibits obvious responses to strain and doping as well. Especially, the slight decoupling between T_MM_ and T_N_ under larger tensile strain (see counterpart on STO) can be observed, while T_MM_ and T_N_ of samples grown in NGO and LAO are roughly close. In other words, such a decoupling usually occurs in the sample under tensile strain compared to compression, similar to the phenomena observed in ultrathin NdNiO_3_ films and superlattices,^[^ ^52^, ^53^ ^]^ which could be attributed to the oxygen octahedral rotation.

**Figure 4:**
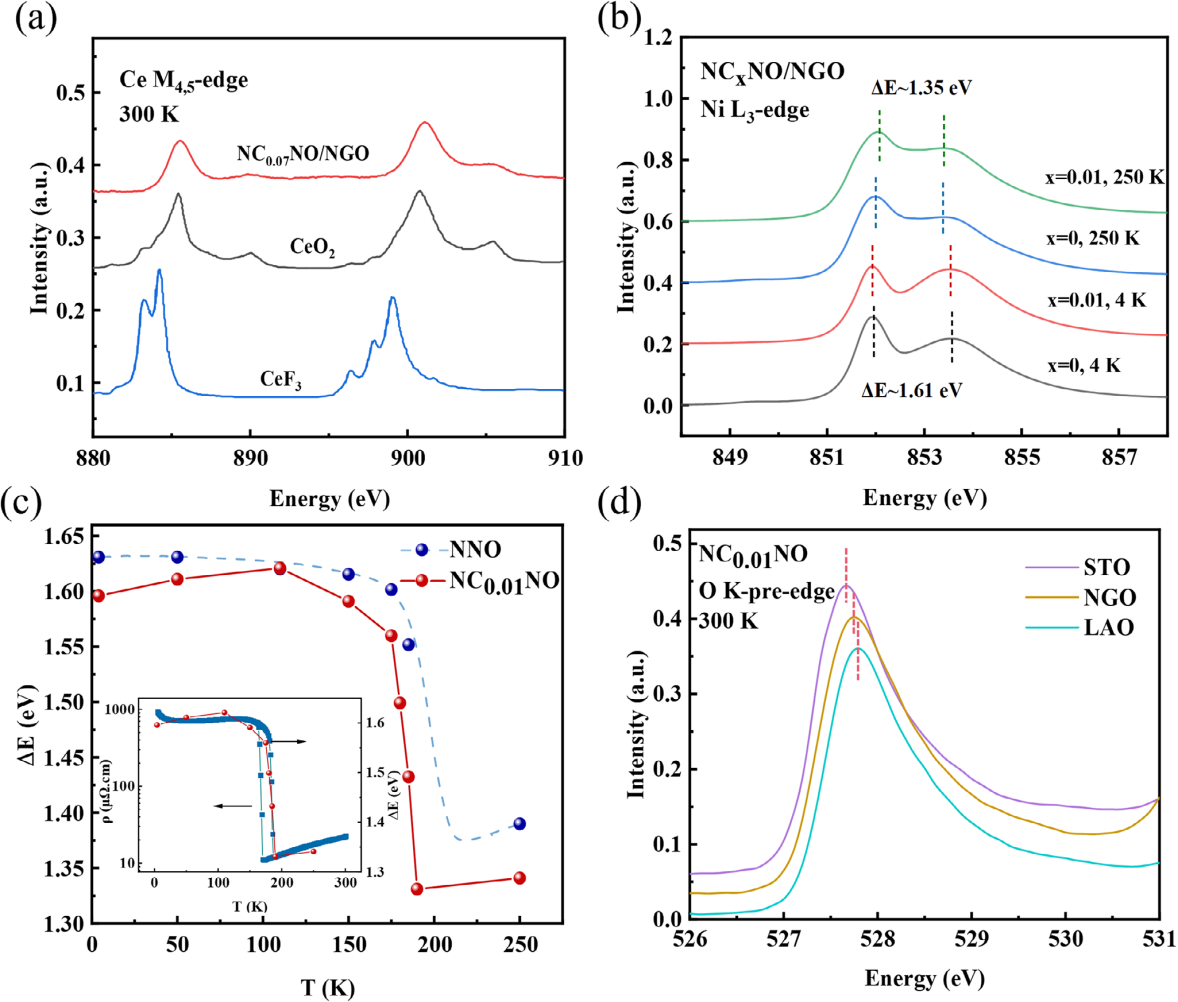
a) Ce M_4,5_‐edge XAS of NC_0.07_NO/NGO, where the experiment spectra of CeF_3_ and CeO_2_ are employed as the references of Ce^3+^ and Ce^4+^, respectively.^[^ ^54^ ^]^ b) The Ni L_3_‐edges XAS of NC_0.01_NO and NNO on NGO substrate at 4 and 250 K, where the gap indicated by the dashed line is defined as ΔE. c) Temperature dependence of the splitting of ΔE. d) Strain dependent of the O K‐pre‐edges of NC_0.01_NO at 300 K, in which the red dushed lines indicate the peak energy.

We further utilized synchrotron X‐ray absorption spectroscopy (XAS) to investigate the evolution of T_MM_ and T_N_ caused by varied substrate strain in the electron‐doped NC_X_NO. **Figure** **4**a exhibits the Ce ‐M_4,5_ edge XAS results. The extremely similar lineshapes between NC_X_NO (x = 0.07) and CeO_2_ reference confirm the Ce valence of +4 rather than +3, which evidences the electron doping in Ce substitution for Nd. The Ni‐L_3_ edge XAS for different Ce‐doped samples at various temperatures are shown in Figure 4b, where the splitting gap, defined as ΔE, can be used to reflect the changes of Ni─O charge‐transfer energy and the emergence of breathing distortion with reducing temperature.^[^ ^42^, ^53^ ^]^ By extracting ΔE for different doping levels at variant temperatures from Figure S6 (Supporting Information), as shown in Figure 4c, a sharp change at 200 K can be observed. The fact that this evolution occurs simultaneously in the metallic NC_X_NO and the insulating undoped NNO suggests that the structure‐driven octahedral breathing distortion does not play a decisive role in the metallic behavior; instead, it could just drive the transition from paramagnetic to antiferromagnetic states.

The pre‐edge of the O‐K edge XAS can be used to reflect the hybridization between the O 2p states and Ni 3d states.^[^ ^42^, ^55^ ^]^ As shown in Figure 4d, the pre‐peak shows a linear correlation with strain. Specifically, as tensile strain decreases or compressive strain is applied, the pre‐peak of the O‐K edge experiences a blueshift. This shift indicates an increase in the concentration of holes in the ligand O^2−^ ions, which can be ascribed to the enhanced Ni─O hybridization or covalency resulting from compression strain.^[^ ^45^, ^56^ ^]^ This, in turn, further leads to a propensity for a shorter Ni─O bond length. Consequently, the Ni─O─Ni bond angle is expected to be straightened, thereby suppressing the orthorhombic to monoclinic structural transition. This explains the paramagnetic metallic ground state observed in NC_X_NO on LAO substrate under compressive strain, as shown in Figure 3c.

## 3 Conclusion

In summary, we have undertaken a systematic investigation to modulate the physical properties of Nd_1‐x_Ce_x_NiO_3_ through the epitaxial strain and controlled Ce doping. Low‐temperature electrical transport measurements, in conjunction with the observed magnetic exchange bias effect, provide unambiguous evidence for the antiferromagnetic metallic ground state in Nd_1‐x_Ce_x_NiO_3_. Furthermore, Ce‐M_4,5_ edge XAS measurements elucidate the +4 valence state of Ce, thereby confirming the electronic doping within Nd_1‐x_Ce_x_NiO_3_. The introduction of the Ce^4+^ dopant effectively suppresses the orthorhombic to the monoclinic phase transition, leading to a hole‐type metallic ground state in Nd_1‐x_Ce_x_NiO_3_ at low temperatures. Notably, epitaxial strain exerts a significant influence on the antiferromagnetic transition temperature, with tensile (compressive) strain exhibiting a positive (negative) correlation. This phenomenon can be attributed to the compressive strain's action in shortening the Ni─O bond length and constraining the deviation of the Ni─O─Ni bond angle from 180°, which is corroborated by the pronounced enhancement of O 2p and Ni 3d hybridization observed in the O‐K edge XAS under compressive strain conditions. Ultimately, it culminates in the successful realization of a transition from an antiferromagnetic metal to a paramagnetic metal state in Nd_1‐x_Ce_x_NiO_3_ (x = 0.05).

It should be pointed out that, although A‐site chemical substitution (whether electron or hole doping) has been shown broadly to suppress the antiferromagnetic ordering temperature in nickelate systems,^[^ ^43^ ^]^ the electron doping (Ce^4+^) has led to the emergence of a metallic state rather than the insulating state typically observed in hole‐doped counterparts.^[^ ^20^, ^57^ ^]^ It is precisely because of the unique electronic/magnetic state of the Ce‐doped counterpart (Nd_1‐x_Ce_x_NiO_3_) that we not only demonstrate the successful engineering of the electronic/magnetic states of antiferromagnetic metallicity through epitaxial strain, but also provide a playground for further exploring emergent phenomena in oxide‐based antiferromagnetic metallic materials.

## Conflict of Interest

The authors declare no conflict of interest.

## Supporting information



Supporting Information

## Data Availability

The data that support the findings of this study are available from the corresponding author upon reasonable request.
