# Supplementary material for: Manipulation of Antiferromagnetic Metal Phase in Nd1‐xCexNiO3 by Epitaxial Strain
Source: Adv Sci (Weinh). 2025 Mar 16;12(18):2415785. doi: 10.1002/advs.202415785 (PMC12079492; doi:10.1002/advs.202415785)
Supplement: Supplementary file 1 — Supporting Information [file ADVS-12-2415785-s001.docx]

Supplementary Information for:

Manipulation of antiferromagnetic metal phase in Nd_1-x_Ce_x_NiO_3_ by epitaxial strain

Zhan Yang^1^, Junhua Liu^2*^, Wen Xiao^2^, Shilin Hu^2^, Zhixiong Deng^2^, Xuedong Bai^3,4^, Lei liao^3,4^, Yulin Gan^2^, Kai Chen^2^, Lifen Wang^3,4^, Zhaoliang Liao^2*^, Haizhong Guo^1，5*^

*^1^School of Physics, Zhengzhou University, Zhengzhou 450001, China.*

*^2^National Synchrotron Radiation Laboratory, School of Nuclear Science and Technology, University of Science and Technology of China, Hefei, 230029, China.*

*^3^Beijing National Laboratory for Condensed Matter Physics and Institute of Physics, Chinese Academy of Sciences, Beijing, 100190, China.
^4^School of Physical Sciences, University of Chinese Academy of Sciences, 100049 Beijing, China.*

*^5^Institute of Quantum Materials and Physics, Henan Academy of Sciences, Zhengzhou 450046, China*

*To whom correspondence should be addressed. E-mail: [hguo@zzu.edu.cn](mailto:hguo@zzu.edu.cn), [zliao@ustc.edu.cn](mailto:zliao@ustc.edu.cn), Liujh0820@mail.ustc.edu.cn.

**MATREIALS AND METHODS**

**Sample preparation**

The Nd_1-x_Ce_x_NiO_3_ films were grown on SrTiO_3_ (001), LaAlO_3_ (001)_pc_ and NdGaO_3_ (001)_pc_ substrates by pulsed laser deposition using a KrF excimer laser (λ=248 nm), with in-situ monitoring by Reflection High Energy Diffraction (RHEED). The laser fluence and repetition rate were 2.3 J/cm^2^ and 3 Hz, respectively. During the deposition process, the substrates were maintained at 600 °C under an oxygen pressure of 0.3 mbar. After deposition, the oxygen pressure was increased to 200 mbar, and the films were cooled to room temperature at a rate of 15 °C/min. The surface morphologies of thin films were characterized by atomic force microscopy (AFM).

**Structure characterization**

X-ray diffraction and reflectivity measurements were performed using a PANalytical Empyrean X-ray diffractometer instrument which is 1.54055 Å for Cu-Kα1 radiation. All films were examined by a high-resolution X-ray diffractometer with both the 2*θ*-*ω* linear scan and off-specular reciprocal space mapping (RSM) mode. The thickness of the films was determined using x-ray reflectivity data, and fitting by GenX.

**Electrical transport measurements**

The temperature-dependent resistivity (*ρ-T*) curves were characterized by a Physical Properties Measurement System (PPMS, Quantum Design) at temperatures ranging from 5 to 300 K. Four-point resistivity measurements were performed in a Van der Pauw geometry with Al wires bonded.

**Anisotropic magnetoresistance (AMR) measurements**.

The effect in antiferromagnets is dependent on the square of the spontaneous magnetization^1^, and the anisotropic magnetoresistance is a dipole function of the magnetization strength^2^. This allows for the observation of similar anisotropic magnetoresistance (AMR) in antiferromagnetic materials as in ferromagnetic materials^1-3^. Consequently, the antiferromagnetic transition temperature (Néel temperature, T_N_) can be inferred from the disappearance of AMR. In Figure S4 (c), we present the angle-dependent AMR of the NC_0.05_NO/STO single film at various temperatures, measured in the [100] plane using a rotating out-of-plane magnetic field (9 T), as illustrated in the inset. At temperatures of 127 K and below, the NC_0.05_NO film displays a two-fold symmetric AMR, indicating magnetic anisotropy characteristic of antiferromagnetic states. With increasing temperature, this AMR diminishes, signifying a transition in the magnetic properties of the NCNO films from antiferromagnetic to paramagnetic states. In Figure S4 (d), we present the temperature-dependent R_max_/R_min_ and determine the antiferromagnetic transition temperature (T_N_) of NC_0.05_NO on various substrates. The T_N_ values for all discussed samples were determined using this approach.

**High-angle annular dark-field (HAADF) measurements**

HAADF images were captured using an aberration-corrected TEM (JEOL Grand ARM 300) operated at 300 kV. The camera length was set to 10 cm, and the collection angles ranged from 54 to 220 mrad. Atomically resolved energy-dispersive X-ray spectroscopy (EDS) maps were recorded using dual EDS detectors. The elemental maps of Ce and Ni were produced according to Ce-M and Ni-K edge, respectively.

**X-ray absorption spectroscopy measurements**

Variable temperature X-ray absorption spectra were measured at Source Optimisee de Lumiere a Energie Intermediaire du LURE in France and MCD-A and MCD-B (Soochow Beamline for Energy Materials) at the Hefei National Synchrotron Radiation Laboratory (NSRL) in China. The experimental spectra presented here were recorded in-situ at 90°incidence angle using the total electron yield (TEY) mode.


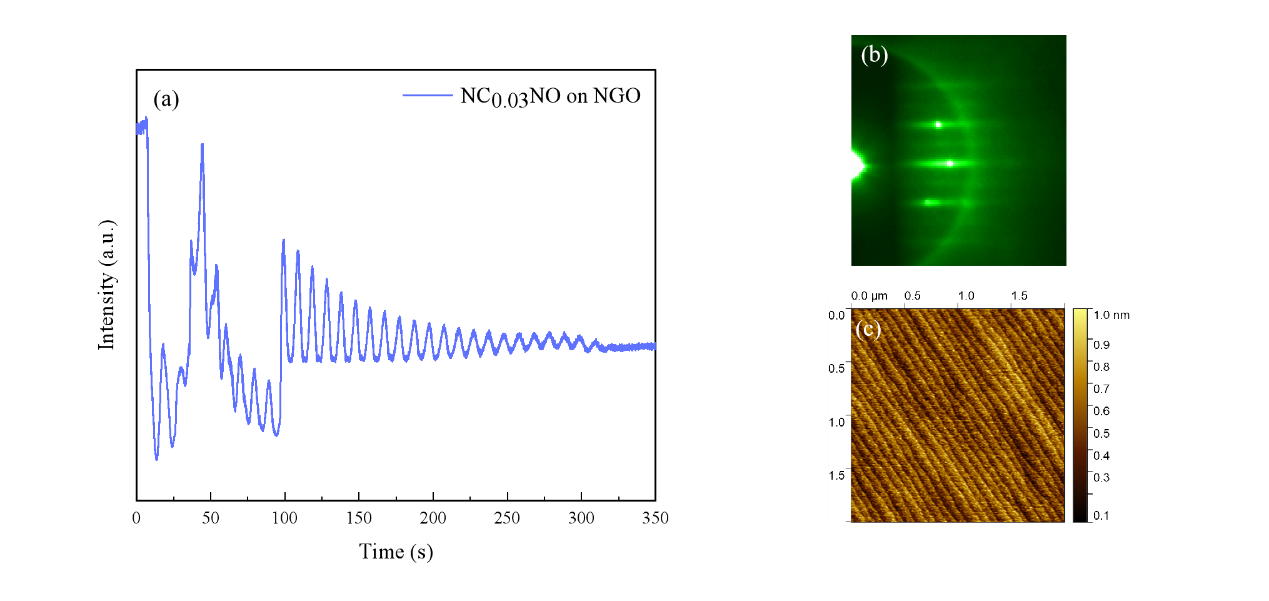


**Figure S1.** Characterization of Nd_1−x_Ce_x_NiO_3_ film synthetized on NdGaO_3_ substrate. (a) In-situ RHEED intensity oscillation of Nd_0.97_Ce_0.03_NiO_3_ film. (b) In-situ RHEED pattern taken after Nd_0.97_Ce_0.03_NiO_3_ film growth on NdGaO_3_ substrate. (c) AFM images of the Nd_0.97_Ce_0.03_NiO_3_.


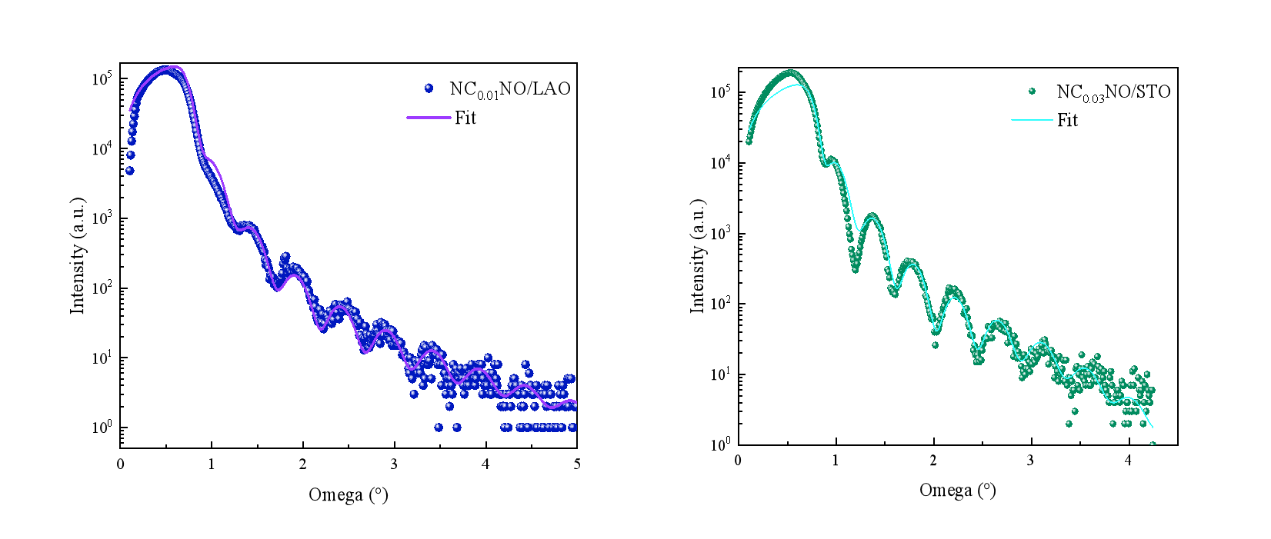


**Figure S2.** XRR fitting for different films growing on different substrates with thicknesses ~15 nm.


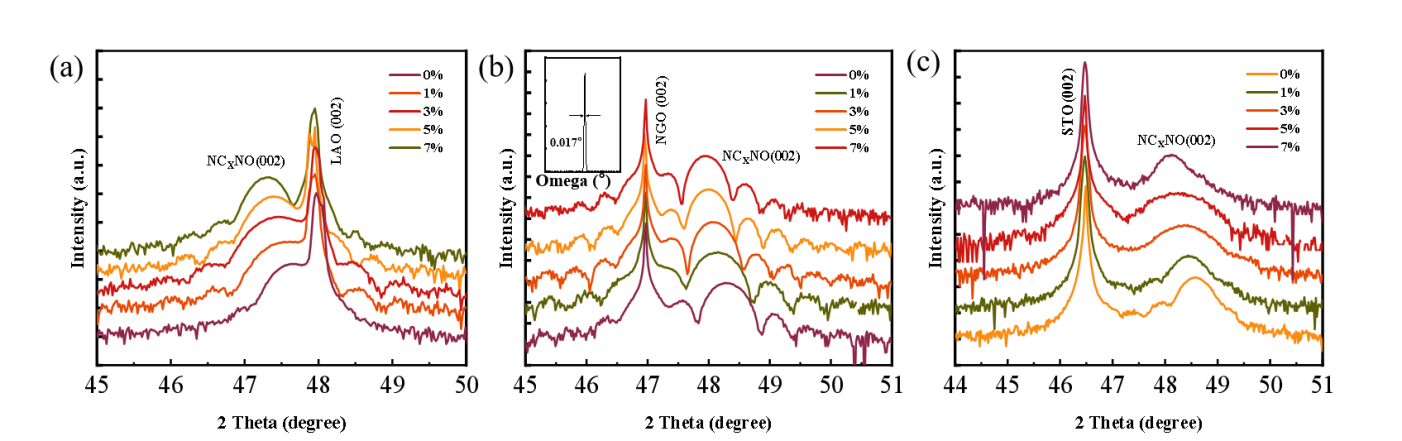


**Figure S3.** (a-c) X-ray diffraction patterns of the NC_x_NO films grown on LAO(001), NGO(001)_PC_ and STO(001). The insets show the rocking curve of NC_0.01_NO on NGO(001)_PC_.


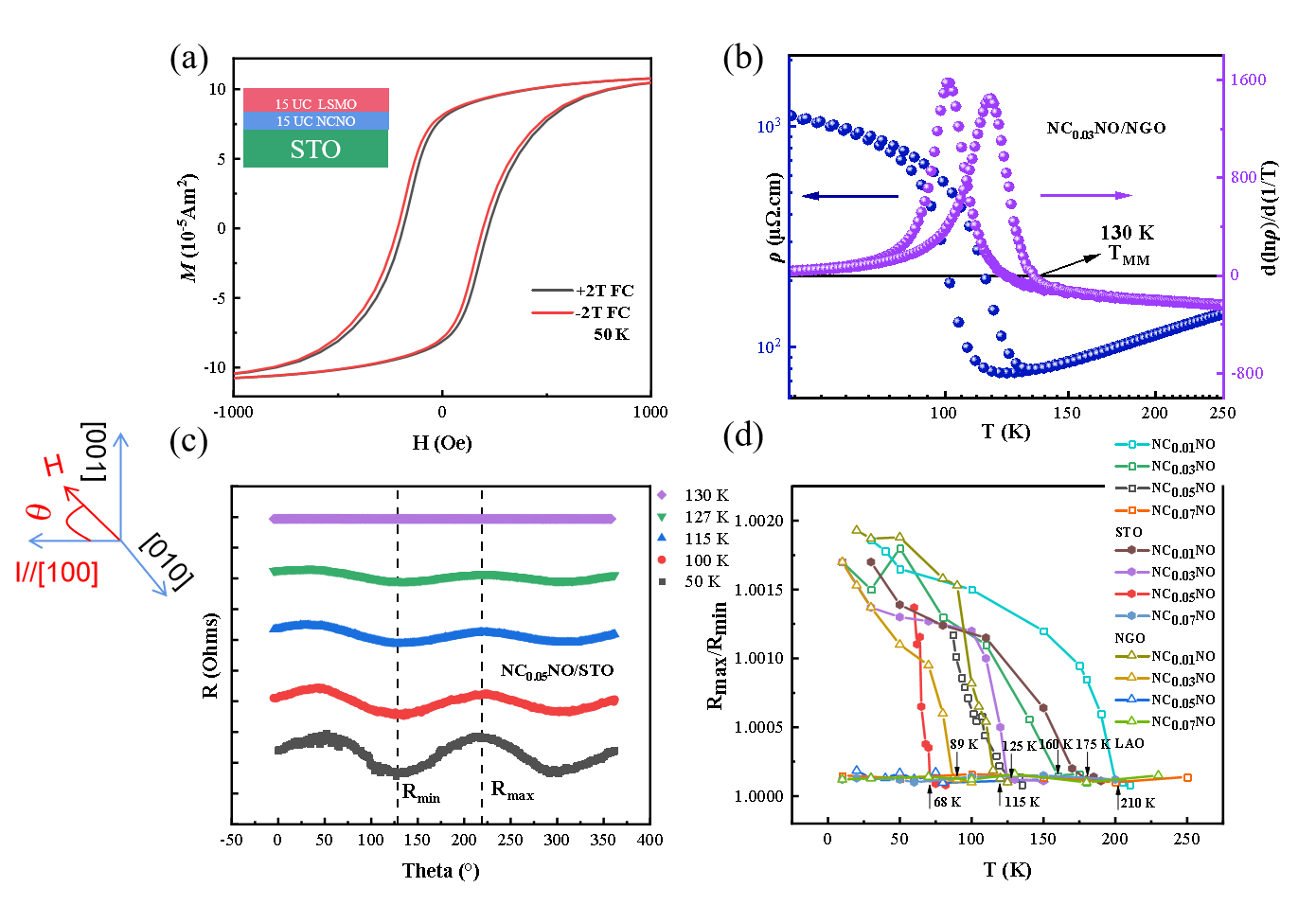


**Figure S4.** (a) M-H curves at 50 K of La_2/3_Sr_1/3_MnO_3_/NC_0.05_NO heterostructures grown on STO under +2T and -2T field cooling. (b) Electrical transport for NC_0.03_NO films grown on NGO. T_MM_ is defined as the temperature at which d (ln ρ)/d (1/T) = 0 in the d(ln ρ)/d(1/T)-T plot. (c) Angle-dependent AMR measured of NC_0.05_NO on STO by a rotating magnetic field (9 T) in the (010) plane during the heating process (θ is the angle between H and the [100] direction). The curves are vertically offset for clarity. (d) temperature-dependent R_max_/R_min_, in which the temperature point that R_max_/R_min_ near to 1 is defined as the T_N_ ^1, 2^.


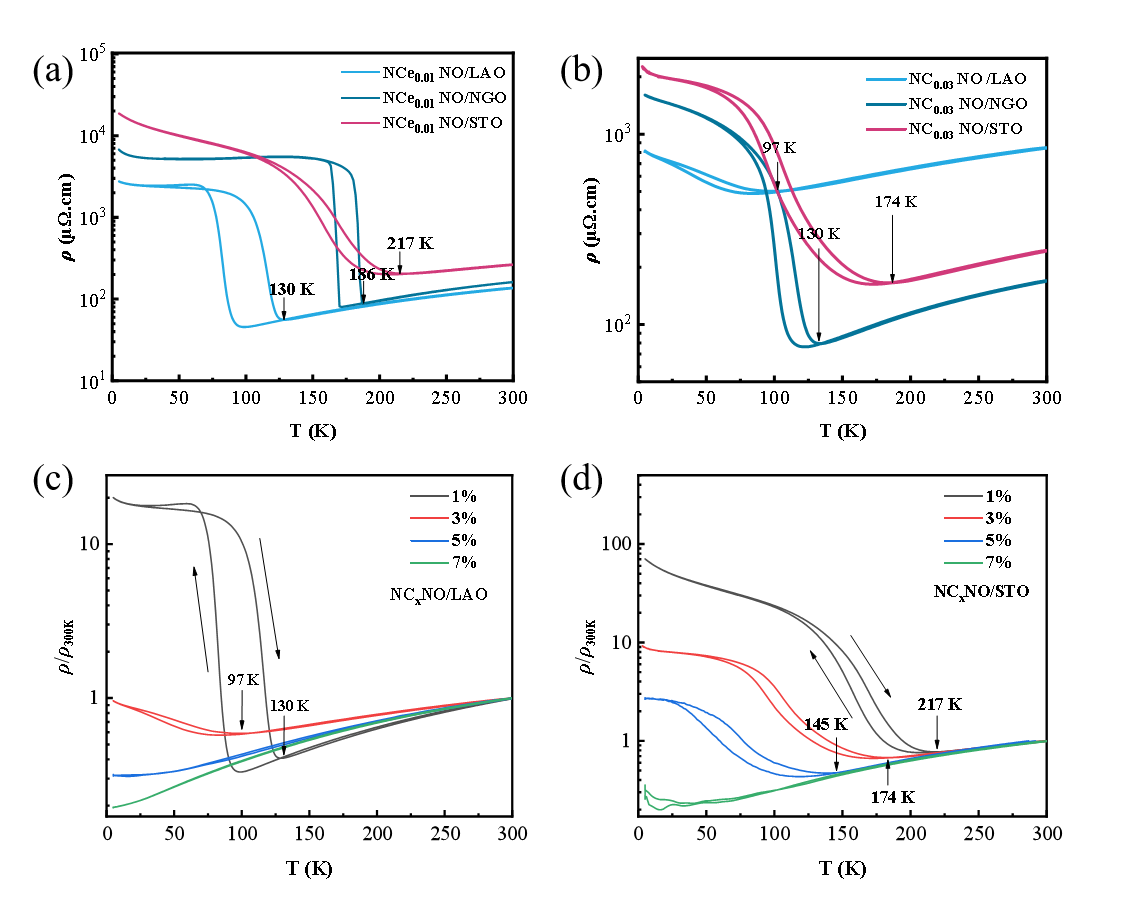


**Figure S5.** (a-b) Electrical transport of the films with the same doping amount of Ce on different substrates. (c-d) Electrical transport of the films with different doping amount of Ce on same substrates.


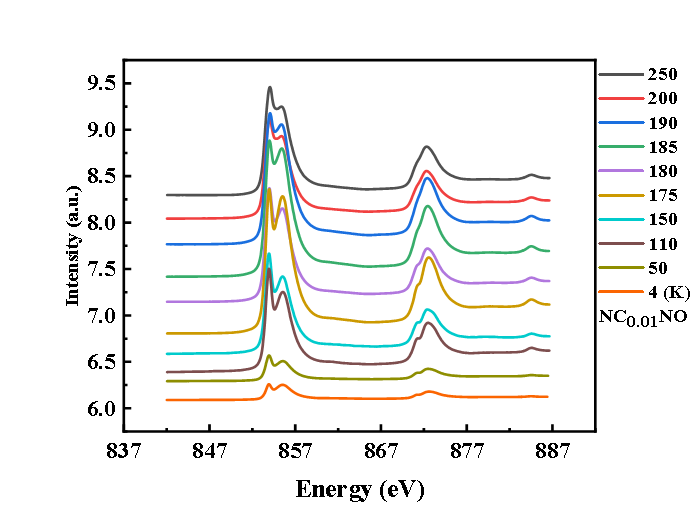


**Figure S6**. X-ray absorption spectra of Ni L-edge of Nd_0.99_Ce_0.01_NiO_3_ on NGO at various temperature.

**Reference**

1. Kriegner, D.; Výborný, K.; Olejník, K.; Reichlová, H.; Novák, V.; Marti, X.; Gazquez, J.; Saidl, V.; Němec, P.; Volobuev, V. V.; Springholz, G.; Holý, V.; Jungwirth, T., Multiple-stable anisotropic magnetoresistance memory in antiferromagnetic MnTe. *Nature Communications* **2016,** *7* (1), 11623.

2. Song, Q.; Doyle, S.; Pan, G. A.; El Baggari, I.; Ferenc Segedin, D.; Córdova Carrizales, D.; Nordlander, J.; Tzschaschel, C.; Ehrets, J. R.; Hasan, Z.; El-Sherif, H.; Krishna, J.; Hanson, C.; LaBollita, H.; Bostwick, A.; Jozwiak, C.; Rotenberg, E.; Xu, S.-Y.; Lanzara, A.; N’Diaye, A. T.; Heikes, C. A.; Liu, Y.; Paik, H.; Brooks, C. M.; Pamuk, B.; Heron, J. T.; Shafer, P.; Ratcliff, W. D.; Botana, A. S.; Moreschini, L.; Mundy, J. A., Antiferromagnetic metal phase in an electron-doped rare-earth nickelate. *Nature Physics* **2023,** *19* (4), 522-528.

3. Nair, N. L.; Maniv, E.; John, C.; Doyle, S.; Orenstein, J.; Analytis, J. G., Electrical switching in a magnetically intercalated transition metal dichalcogenide. *Nature Materials* **2020,** *19* (2), 153-157.
